# Evaluation of Two Web-Based Interventions (REMOTION and Res-Up!) for Clients From Psychotherapy Waitlists in Routine Outpatient Psychotherapy (Therapy Online Plus-TOP): Randomized Controlled Trial

**DOI:** 10.2196/83917

**Published:** 2026-07-08

**Authors:** Leonie Franziska Trimpop, Laura Luisa Bielinski, Jan Schürmann-Vengels, Sebastian Appelbaum, Thomas Berger, Ulrike Willutzki

**Affiliations:** 1 Department of Psychology and Psychotherapy, Witten/Herdecke University, Alfred-Herrhausen-Strasse 50, Witten, 58448, Germany, 49 2302-926-9704; 2 Department of Clinical Psychology and Psychotherapy, University of Bern, Bern, Switzerland

**Keywords:** randomized controlled trial, RCT, internet-based therapy, transdiagnostic, resilience, emotion regulation, waitlist

## Abstract

**Background:**

Internet-based interventions can improve treatment accessibility, prevent chronicity, and reduce waiting times. Despite their potential, the effectiveness of transdiagnostic internet-based interventions for individuals on waiting lists, and their integration into routine care, remains insufficiently evaluated.

**Objective:**

This study aimed to compare 2 transdiagnostic web-based interventions focusing on improving emotion regulation and resilience to a control group (CG) in individuals recruited from waiting lists for routine outpatient psychotherapy.

**Methods:**

At 4 outpatient centers in Germany, 421 adult participants were recruited from respective waiting lists and randomized into 1 of 2 intervention groups (ie, “REMOTION” and “Res-Up!”) or the CG without access to the interventions. Participants were not restricted from accessing face-to-face psychotherapy during the course of the study. Assessments occurred at baseline, 6 weeks after intervention, and at 12-week follow-up. The primary outcome was symptom severity. The secondary outcomes focused on emotion regulation and resilience. Other outcomes included depressive symptoms, self-compassion, and self-esteem.

**Results:**

No significant differences were found between the intervention groups and the CG for the primary outcome symptom severity, although all groups significantly improved over time. The secondary outcomes for emotion regulation improvement found significant between-group differences only when comparing REMOTION to CG in acceptance of negative emotions. For resilience and all other outcomes, most groups improved significantly over time, but treatment groups did not differ from CG.

**Conclusions:**

Although we were able to find significant improvements across time for the treatment groups, participants in the CG improved significantly as well. Findings for between-group interactions show small additional benefits for participation in REMOTION for emotion regulation compared to the CG only. The study provides information for future research concerning the effectiveness of and difficulties in implementing transdiagnostic internet-based interventions during waiting times for psychotherapy. Our findings suggest that transdiagnostic internet-based interventions do not seem to be more effective than simply waiting for psychotherapy.

## Introduction

### Background

Approximately 1 (17.6%) in 6 people worldwide met the criteria for a mental disorder, and nearly one-third (29.2%) had experienced a mental disorder at some point in their lives [1]. Mental health concerns and disorders have a profound negative impact on individuals affected by symptoms and, therefore, on society as a whole. In Germany, despite clinical need, only 11% to 40% of patients receive appropriate treatment depending on various factors, indicating substantial gaps in psychotherapy care access and contribution [2,3]. The most common reasons are long waiting times for psychotherapy (eg, 20.1 weeks in 2019 [4]), fear of stigmatization, or barriers inherent to the type of disorder (eg, social anxiety and suicidality [5] or depression [6]).

Interventions provided via apps or web are an innovative way of delivering much-needed treatments and can thereby help in closing the earlier mentioned psychotherapy care gap [7-9]. With limited access to psychotherapy, online interventions are an economical, flexible, and practical addition to conventional treatment [8]. Particularly, when face-to-face psychotherapy is not available due to various types of barriers (such as insufficient availability of therapists or fear of facing stigmatization), internet-based interventions may help to avoid long waiting times for treatment and thus mitigate the chronification of mental health problems [7]. Research on internet-based self-help interventions in psychotherapy demonstrates the effectiveness of different intervention programs for a variety of mental disorders [8,10,11]. Different types of internet-based interventions can function as stand-alone online programs or be conceptualized as blended treatments—the combination of face-to-face psychotherapy with online interventions [12].

Concerning internet-based interventions during waiting times for psychotherapy, a recent review of Huang et al [13] found only 5 randomized controlled trials (RCTs) and 3 uncontrolled studies focusing on depression and anxiety. While these studies showed no additional benefit for internet-based interventions compared to waiting lists, the authors concluded that more high-quality RCTs with larger sample sizes are warranted. Some studies, however, show incremental benefits for internet-based psychotherapy over waiting controls with varying effect sizes [14-17]. In particular, transdiagnostic internet-based interventions might be a promising approach as they are suitable for patients with a wide spectrum of mental health problems and comorbid disorders can be addressed [16,18]. Furthermore, universally tailored transdiagnostic treatments may be more effective than tailored ones [19]. When looking at psychotherapy interventions in general, a differentiation between interventions focusing more on compensation and those focusing more on capitalization is possible. Compensation-oriented interventions identify dysfunctional maintenance factors of psychopathology and teach or train the person to use new strategies or build new behavior against relative deficits, whereas capitalization-oriented interventions aim to draw on the person’s already existing strengths (eg, action repertoire, resilience strategies, and external as well as internal resources) [20-24]. Research indicates that focusing on strength-based approaches (including capitalization) in psychotherapies is beneficial for therapy outcomes, especially with regard to well-being [25-27]. Compensation-oriented interventions might be needed during waiting times to counter dysfunctional maintenance factors of psychopathology, but when therapeutic support is limited, it might be particularly important to activate and support a person’s strengths and thus follow a capitalization-oriented approach. Research on compensation and capitalization interventions, especially including control groups (CGs), is difficult and sparse [20,23,28-31]. To increase knowledge concerning said concepts, we set the emotion regulation intervention as more compensation focused and the resilience improvement intervention as more capitalization focused, as both concepts have been shown to be effective methods in psychotherapy [32].

Thus, this RCT explores, for the first time, the effect of 2 transdiagnostic web-based interventions delivered via an internet browser compared to a care-as-usual–only CG in routine outpatient care.

### Emotion Regulation (as the More Compensation-Oriented Approach)

The concept of emotion regulation is relevant to the treatment of a wide range of mental health disorders [33-37]. Different definitions of emotion regulation exist in the literature. According to Gross [38], emotion regulation is the process by which individuals influence which emotions they have, when they have them, and how they experience and express them. Several web- and mobile-based mental health interventions have been developed to target this construct [39,40]. Using the extended process model of emotion regulation that differentiates between the identification, selection, and implementation phases of emotion regulation as a framework [41], the web-based REMOTION intervention was developed [42]. The internet-based program used in this trial aims to improve patients’ emotion regulation and thus reduce their symptom severity. The program has been tested in an outpatient psychotherapy setting in a pilot RCT [43] and also as an add-on to acute inpatient psychiatric care in a pilot RCT [44,45].

### Resilience (as the more Capitalization-Oriented Approach)

Resilience and resources or strengths are often discussed in the field of positive psychology [46], focusing on factors increasing health rather than decreasing symptoms. Most research on resilience aims at building new strengths [47]. However, the evidence-based personal model of resilience (PMR), developed by Padesky and Mooney [48] from a cognitive perspective, takes a clear capitalization approach: The PMR emphasizes individuals’ preexisting strengths and resilient strategies, aiming to enhance awareness and the use of these strengths. Its basic premise is that everyone possesses resilience strategies that are already part of their behavioral repertoire and can be used in challenging situations. The PMR is grounded in evidence-based standards for cognitive behavioral therapy [49] and has proven to be helpful in several studies [50-52]. In this study, Res-Up! (Resilienz – unkompliziert und persönlich!) is implemented as a web-based version of the program to foster resilience in participants. The internet-based version of the PMR “Res-Up!” (resilience program of the University of Witten/Herdecke) as a stand-alone program without a CG showed significant improvements over time, with medium effect sizes in improving resilience (*d*=0.51-0.55) and emotional competence (*d*=0.51), greater effect sizes for self-compassion (*d*=0.70), and small effect sizes for self-esteem (*d*=0.41) [53].

### Aims

Many controlled studies have shown the efficacy of internet-based interventions. There is still limited evidence about the effectiveness of transdiagnostic approaches for internet-based interventions in routine psychotherapeutic practice and specifically their usefulness in bridging the gap between no support and the beginning of psychotherapy, especially during waiting times for therapy [13]. In this study, the *goal* was to assess the effectiveness of an emotion regulation improving intervention (REMOTION, more compensation oriented) and a resilience improving intervention (Res-Up!, more capitalization oriented) web-based intervention in an outpatient psychotherapeutic routine setting in comparison to a care-as-usual–only CG. In accordance with previous research on internet-based interventions as stand-alone, blended care and compared to care-as-usual and waiting conditions [14,15,54,55], we hypothesized that both interventions were more efficacious than no additional treatment. The primary outcome targets overall symptom severity. The secondary outcomes assess improvement of emotion regulation and resilience as the specific target areas of the individual interventions, whereas other outcomes target depressive symptoms, self-esteem, and self-compassion.

## Methods

### Study Design

In this 3-arm multicenter RCT, 421 participants were randomized into 1 of 2 web-based interventions (“REMOTION” and “Res-Up!”) or a CG. Three assessments were conducted: baseline, after 6 weeks (post), and after 12 weeks (follow-up) for all participants. Participants in the intervention groups received access to the respective intervention immediately, whereas participants in the CG were given access to one or both interventions of their own choice after 12 weeks, that is, completion of the last assessment. Participating centers of the trial were the outpatient clinics of the Training Center for Psychological Psychotherapy OWL (Bielefeld, Germany), the Centers for Psychotherapy Dortmund and Münster (all training centers of the German Association for Behavior Therapy-Deutsche Gesellschaft für Verhaltenstherapie, DGVT), and the Center for Mental Health and Psychotherapy (Department of Psychology and Psychotherapy, University Witten/Herdecke, Germany).

### Eligibility Criteria

Inclusion criteria were (1) current diagnosis of a mental disorder according to the *ICD-10* (*International Statistical Classification of Diseases, Tenth Revision*); (2) recruitment at one of the participating outpatient clinics listed earlier; (3) interest to receive psychotherapy at one of the centers, but not currently enrolled; (4) aged at least 18 years; and (5) reliable internet access. Exclusion criteria were (1) current severe episode of major depression; (2) current psychotic disorder; (3) acute suicidal tendency; (4) other severe mental disorders, for example, current alcohol or substance abuse or current bipolar disorder; and (5) insufficient German language skills. Diagnostic criteria were assessed via a clinical assessment or structured clinical interview. Access to face-to-face psychotherapy after enrollment was not restricted while participants were working with the programs, mirroring naturalistic conditions.

### Recruitment, Randomization, and Trial Organization

Participant recruitment and data collection started in April 2020 and ended in July 2022 (thus mostly during COVID-19 lockdowns in Germany). Participants were recruited during registration to regular psychotherapy in the described outpatient centers in Germany. Recruiting outpatient centers received information about the study from a member of the study team [56]. Information on the study and the interventions (REMOTION and Res-Up!) was given to interested individuals coming to the institution for a face-to-face consultation session with additional written information by trained psychotherapists at all centers. Given the long waiting times for psychotherapy in Germany (ranging from 3 mo to 1.5 y), the consultation generally does not mark the beginning of psychotherapy but is scheduled to evaluate whether the person has a clinically relevant psychiatric disorder, and clients are added to waiting lists for psychotherapy afterward. All participants were required to give their informed written consent before enrollment in the study, which was solicited by the respective practitioner and handed to one specified member of the study team, including necessary information (name, birthdate, diagnoses, date of recruitment, and email). After proving study eligibility, participants were sent an email from the study team with an individualized code to the baseline measure via Qualtrics software, using the current version during the period of data collection, 2020-2022 [57,58]. Answering individual questionnaires took approximately 30 minutes at every assessment point. After baseline completion, participants were randomly assigned by a randomized computer generator (in Qualtrics [57]) to 1 of the 3 treatment arms. As completion of the interventions took approximately 6 weeks, posttreatment measures were provided 6 weeks after baseline. Follow-up measures were provided to participants 12 weeks after baseline; follow-up was set this close to the post measure to reach participants with minimal overlap with face-to-face psychotherapy. Participants in the CG were informed that access to the programs would be granted after completing follow-up. In the experimental conditions, participants received an email with their online access to either (1) REMOTION (focusing on emotion regulation) or (2) Res-Up! (focusing on resilience) according to their randomly assigned group. Participants were given the option to contact a member of the study team via text messages during the intervention (“guidance on demand” and “minimal human guidance” [59]) but were not actively contacted by the team (excluding reminders to complete the interventions or questionnaires and a welcome message). Ultimately, 421 participants were randomized into 3 arms with a 1:1:1 allocation ratio (CG: n=141, REMOTION: n=141, and Res-Up!: n=139). Participants in the sample showed a variety of different mental disorders with varying symptom severity, which matches the transdiagnostic approach of the interventions.

### Sample Size Calculation

G*Power, current version before the period of data collection 2020 (G*Power is distributed from this web page [60]), [61] was used to calculate the sample size. We planned to detect small effect sizes of Cohen’s *d* of 0.20 (respectively, Cohen’s *f*=0.1) regarding the time×group interaction for the 2 active conditions, powered separately, compared to the CG at a Bonferroni-corrected α error level of 0.006. According to the power analysis, a sample size of 103 participants in each of the 3 study arms was required to detect a statistically significant difference with a power (1−β) of .80. Results differ from the study protocol due to the previously uncorrected α level. Due to the high dropout rate (54.3% instead of the estimated 25%), which occurred early in the study during COVID-19 lockdowns, the number of recruited participants was increased during the course of the study to allow additional per-protocol analyses. Dropout rates from internet-based interventions are a frequently reported problem [54,62,63]. We tried to accommodate for this initially by choosing an at least minimally guided format, thus giving participants the possibility to contact the study team via text messages throughout the whole study, which should have increased adherence to the internet-based programs [64].

### Interventions

#### Overview

Participants of both interventions were asked to complete 1 module per week. A variety of psychotherapy elements (eg, cognitive behavioral therapy and positive therapy) were integrated in the program, including video, text, and audio, along with different types of exercises. Participants were reminded to continue working if they had not used the intervention for an entire week but received no other active guidance component (such as weekly emails). However, participants were able to contact the study team via text messages, if any questions occurred (guidance on demand). To increase adherence, reminder messages to complete the modules on time were sent. Completion of both programs was intended to take approximately 6 weeks.

#### REMOTION as Compensation-Oriented Intervention

**Figure 1.**
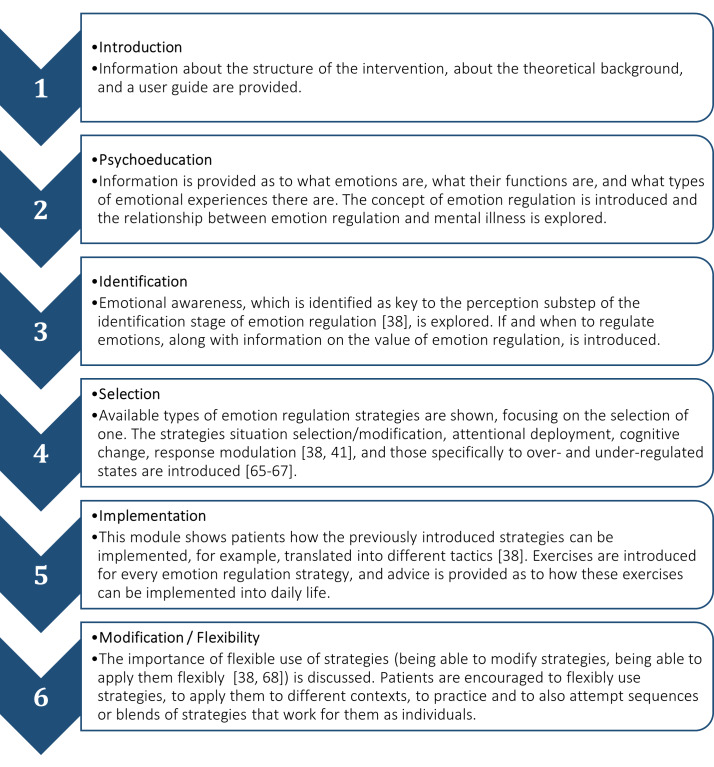
Contents of REMOTION (adapted from [42]) [38,41,65-68].

REMOTION is a web-based intervention, with password-protected access managed and provided by the University of Bern (Switzerland). A detailed theoretical background for the intervention can be found in the study by Bielinski et al [42]. The goal of the intervention is to improve emotion regulation and reduce patients’ symptom severity. Participants first complete an introductory module and then work on 1 of 5 modules per week; completing a module takes approximately 1 to 2 hours. Figure 1 shows the modules and content of the intervention.

#### Res-Up! as Capitalization-Oriented Intervention

**Figure 2.**
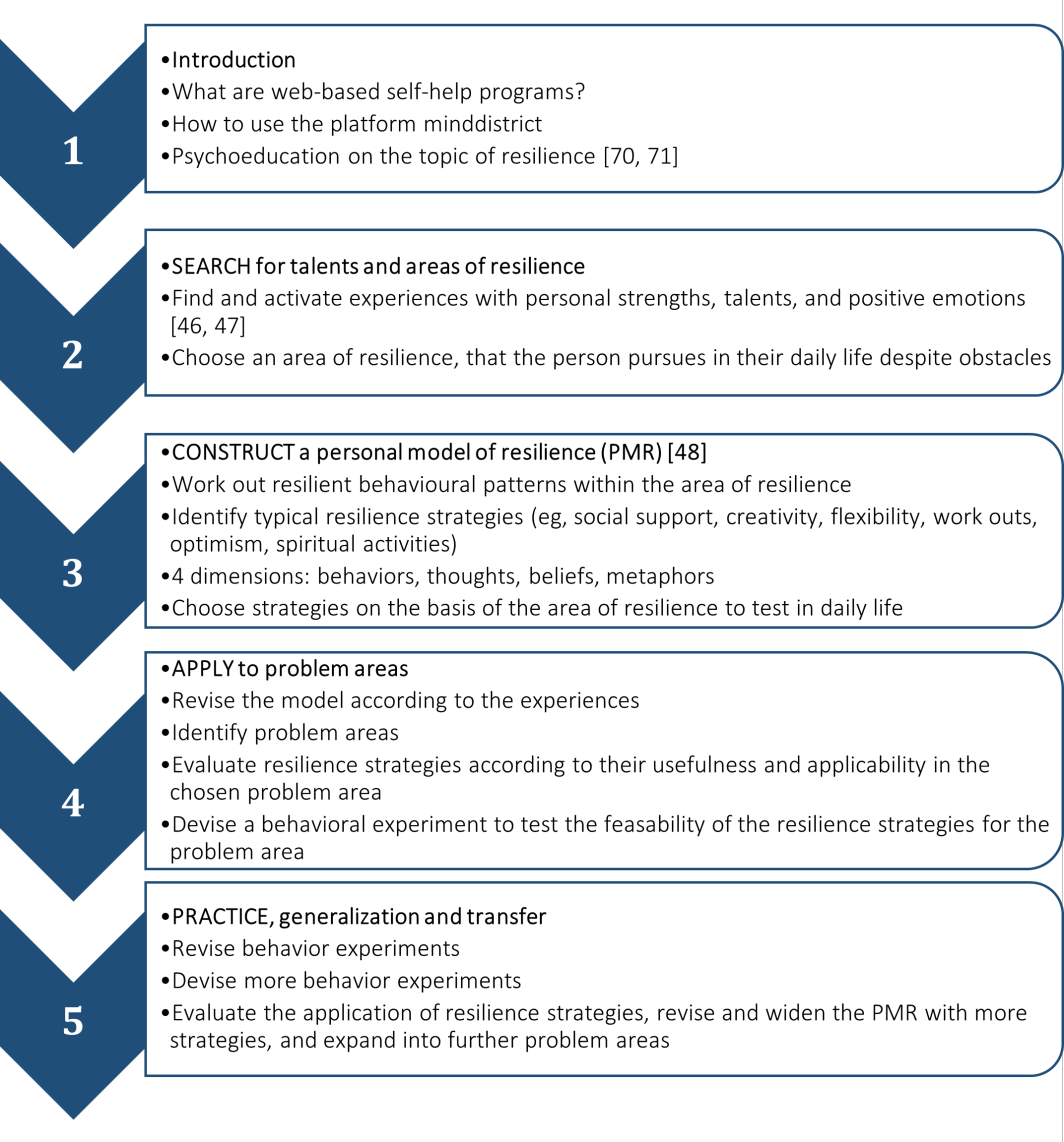
Contents of Res-Up! [46-48,70,71].

Res-Up! is a web-based intervention focusing on patients’ strengths and positive experiences, developed and managed at the University Witten/Herdecke (Germany) and provided via Minddistrict [69]. It is based on the PMR [48], which is a positive cognitive intervention that uses patients’ existing strengths to overcome problems. Participants work on 1 of 5 consecutive modules (Figure 2) per week; completing a module takes approximately 1 to 2 hours. A detailed description of Res-Up! can be found in the study protocol [56].

#### Control Group

Participants in the CG answered the questionnaires before randomization as well as 6 and 12 weeks later. Access to the web-based intervention of their choice was given after completing the 12-week follow-up assessment. During the waiting time, participants were allowed to stay on the wait list or start psychotherapy as they would usually do.

### Measures

#### Overview

Demographic information of participants was recorded at baseline. Information concerning additional psychotherapy received by participants during the course of the study was gathered at baseline, 6 weeks, and 12 weeks. Participants were asked if they were currently attending psychotherapy sessions (“yes” or “no”). Patient’s diagnostic status was obtained by an experienced psychotherapist at the respective outpatient clinics during the initial interview, either by conducting a structured clinical interview I (German version) for diagnostic [72,73] or clinical assessment, depending on the center’s routine practice. Outcomes were gathered online via link to online self-report questionnaires based on Qualtrics software [57]; for a detailed overview, see Table A in Multimedia Appendix 1. Data collection was supported by email reminders. Irrespective of whether the participant started parallel face-to-face treatment or the state of completion of the program, assessments occurred at the given time points. Participants received weekly reminders via email, up to a maximum of 3, if they did not fill out questionnaires.

#### Primary Outcome Measure

*General symptom severity,* measured with the Brief Symptom Inventory-short form (BSI-18; German version [73], 2011) of the SCL-90-Revised (SCL-90R [74]), was the primary outcome measure. The BSI-18 consists of 18 items and is a frequently used questionnaire to measure general symptom severity, with 3 subscales: somatization, depression, and anxiety (Cronbach's α=0.85-0.89), with good psychometric properties [75], comparable to those of the SCL-90R [74]. In our sample, Cronbach's α ranged from 0.88 to 0.91.

#### Secondary Outcome Measures

*Emotion regulation* was assessed via the following two instruments: (1) self-assessment of emotion regulation skills (SEK-27; Fragebogen zur standardisierten Selbsteinschätzung emotionaler Kompetenzen [76]), a German 27-item self-report measure of emotion regulation skills with high reliability and validity (Cronbach's α=0.90; in our sample=0.93-0.95); and (2) questionnaire assessing the acceptance of unpleasant and pleasant emotions (FrAGe; Fragebogen zur Akzeptanz von Gefühlen [77]), a German 32-item self-report measure of acceptance and suppression of pleasant and unpleasant emotions with good reliability and validity. In our sample, Cronbach's α was 0.93-0.95.

*Resilience* was assessed via the following two instruments: (1) Witten Strengths and Resource Form (WIRF [78]), a German 37-item self-report of personal and external strengths with high reliability (Cronbach's α=0.84-0.88; in our sample=0.91-0.94); and (2) the Connor-Davidson Resilience Scale (CD-RISC-10 [79]), an internationally used 10-item self-report of individual resilience with high reliability (Cronbach's α=0.81-0.90; in our sample=0.84-0.88).

*Severity of depression* was assessed with the German version of the Patient Health Questionnaire-9 (PHQ-9 [80]). The PHQ-9 is an internationally used 9-item self-report for screening, diagnosing, monitoring, and measuring the severity of depression with a high retest reliability and validity (Cronbach's α*>*0.86; in our sample=0.81-0.86).

*Self-esteem* was assessed with the Rosenberg-Self-Esteem Scale (RSES [81]), an internationally used 10-item self-report of general self-esteem with high reliability and validity (Cronbach's α*=*0.72-0.85; in our sample=0.91-0.92).

*Self-compassion* was assessed with the Self-Compassion Scale (SCS-D [82]), an internationally used 26-item self-report of self-compassion with high reliability and validity (Cronbach's α>0.90; in our sample=0.90-0.93).

The *therapeutic alliance* between participants and the web-based intervention or study team was measured with an adapted version of the Working Alliance Inventory for guided internet interventions (WAI-I [83]) at the end of the intervention (6 wk) and at follow-up (12 wk). The WAI-I is a 12-item self-report scale. For the purpose of this study, the term “psychologist” was substituted with the term “study team” in the questionnaire. The WAI-I has good internal consistency at total and subscale level (Cronbach's α between 0.92 and 0.94; in our sample=0.92-0.94).

### Statistical Analyses

Data were analyzed using an intention-to-treat (ITT) approach, including all randomized patients in the outcome analyses (Figure 2), using R Statistical Software (version 4.3.2; R Core Team 2021 [84]) and SPSS (version 29; IBM Corp). For categorical data, counts or percentages were reported. Effect sizes of all between-group and within-group pre-to-follow-up changes were computed as Cohen’s *d* [85], using an ITT approach unless stated otherwise. In addition, reliable change indices (RCIs [86]) of changes were analyzed by subtracting T1 means from T3 means of every participant, divided by the SE of differences between the 2 scores of the respective (sub)scales [86]. Scores below 1.96 were considered as relevant decreases, whereas scores above 1.96 were considered as relevant increases. Depending on the questionnaire, this indicated improvements or reductions in the respective factors.

Effects of the intervention and CGs on the primary and secondary outcome measures across time were analyzed with multilevel models (MLMs). MLMs are recommended for ITT analyses with missing data because of the possibility to accommodate for larger amounts of missing data without having to exclude or impute data, not depending on limited assumptions about the variance-covariance matrix. Previous research showed that the power of MLM is comparable to (or even higher than) power of ANOVA designs [87-89].

**Figure 3.**
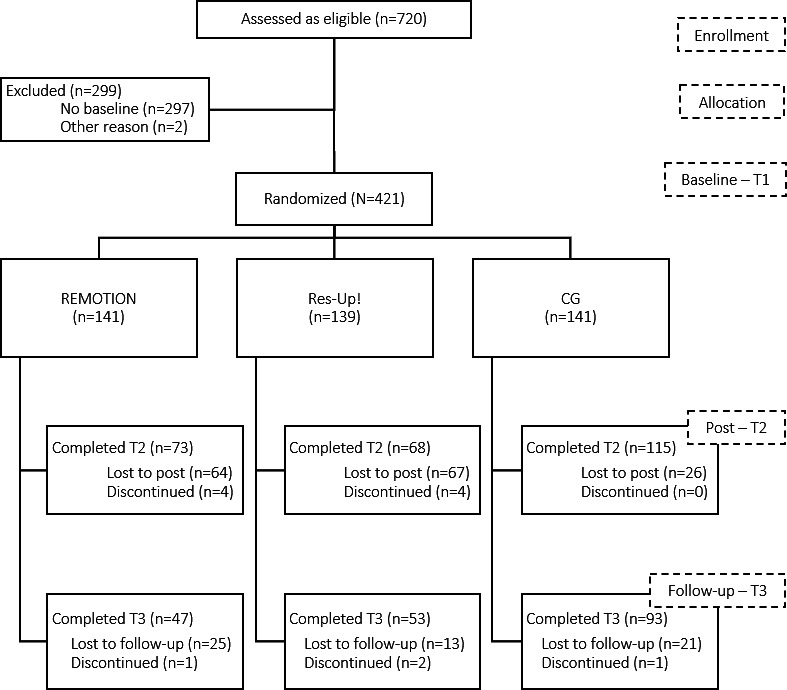
Flow of patients through the study. CG: control group.

MLMs were used to observe changes in symptom severity (primary); emotion regulation and resilience (secondary); and depressive symptoms, self-esteem, and self-compassion (other outcomes). Three-level models were used, considering the repeated assessments (level 1) as nested within patients (level 2), nested within locations (level 3). The base model 0 was calculated using random intercepts with fixed slopes on the patient level, and no predictors were included. Pseudo*-R²* was used as a measure of effect size for level 1 [90], which represents the amount of explained level 1 variance, that is, the amount of within-patient fluctuations in the respective main predictor, accounted for by the respective model. The intraclass correlation coefficient was used as a measure of the proportion of variance for the random effect within level 2 and can be interpreted as the expected correlation between 2 randomly drawn level 1 units within a given randomly drawn level 2 unit [91]. The magnitude of this measure can be interpreted similarly to a correlation coefficient, thus representing an effect size index [91,92]. Model fit of competing models was compared by means of likelihood ratio tests for nested models and the Akaike Information Criterion, with smaller values indicating a better fit. Time was coded 0, 6, and 12, assuming a linear trend with realistic intervals according to the measurement points at baseline, 6 weeks, and 12 weeks, and included as the main predictor in model 1. Between-group effects were added in model 2 for the 3 arms (REMOTION, Res-Up!, and CG) to control for variance between the groups before treatment. In the final model 3, we added the interaction time×group as the main predictor to observe possible differences in the change of the respective outcomes between the groups across time. Simple slope analyses were conducted to examine within-group changes over time for each outcome. For additional effect size calculation in the case of MLMs, we used a Cohen’s *d* equivalent [93], which was calculated by multiplying the difference in the rate of change in the outcome between 2 groups per unit of time (*b*) by the number of time points minus 6 (as time was coded 0, 6, and 12) (*duration*) and dividing that by the pooled SD of the 2 respective groups of the means of the raw scores at baseline (either REMOTION and CG or Res-Up! and CG) [94]. As this is a Cohen’s *d* equivalent, effects are considered small at 0.20, medium at 0.50, and large at 0.80 [85]. A Bonferroni-corrected α level of 0.006 was applied to adjust for multiple (9 measurement instruments overall) comparisons [95]. Results were reported in accordance with CONSORT (Figure 3; Consolidated Standards of Reporting Trials; [96]) and CONSORT-EHEALTH (Consolidated Standards of Reporting Trials of Electronic and Mobile Health Applications and Online Telehealth) checklists [97].

### Ethical Considerations

The study was conducted according to local regulations and the Declaration of Helsinki. The study was approved by the ethics committee of the University Witten/Herdecke (221/2019). Written informed consent regarding the gathered data and following analyses as well as scientific publications in an anonymous form was obtained from all patients by the recruiting therapists in outpatient centers. Neither recruiting therapists nor patients received compensation in any form. Participating patients gained access to the treatments for free (CG after 12-wk follow-up measure completion). The trial was preregistered with ClinicalTrials.gov (NCT04352010). A study protocol was published prior to the study [56]. Data were only analyzed in an anonymous form and deidentified by a member of the research team.

## Results

### Sample Characteristics

Groups did not differ concerning demographics and diagnosis, comorbidity, and medication at baseline (Table 1). Participants showed a wide variety of mental disorders and comorbidity, with depression being the most common diagnosis (48.46%). Figure 3 displays the flow of patients through the study.

**Table 1.** Demographics of all randomized participants.

Characteristics	Entire sample (N=421)	REMOTION (n=141)	Res-Up! (n=139)	CG^a^ (n=141)
Age (y), mean (SD)	36.50 (13.01)	35.36 (13.30)	37.55 (12.81)	36.57 (12.91)
Sex^b^, n (%)				
Female	270 (64.1)	95 (67.4)	86 (61.9)	89 (63.1)
Male	148 (35.2)	45 (31.9)	52 (37.4)	51 (36.2)
Other	3 (0.7)	1 (0.7)	1 (0.7)	1 (0.7)
Nationality^c^, n (%)				
German	402 (95.5)	138 (97.9)	128 (92.1)	136 (96.5)
Other	19 (4.2)	3 (2.1)	11 (7.7)	5 (3.5)
Relationship status^d^, n (%)				
Single	181 (43.0)	64 (45.4)	59 (42.4)	58 (41.1)
In a relationship	198 (47.0)	64 (45.4)	65 (46.8)	69 (49.0)
Separated	42 (10.0)	13 (9.2)	15 (10.8)	14 (9.9)
Employment^e^, n (%)				
Employed	240 (56.9)	80 (56.7)	83 (59.7)	75 (53.2)
Unemployed	43 (10.2)	11 (7.8)	16 (11.5)	16 (11.3)
Student or in training	106 (25.1)	34 (24.1)	30 (21.6)	43 (30.5)
No answer	32 (7.6)	16 (11.4)	10 (7.2)	7 (5.0)
Education^f^, n (%)				
University	156 (37.1)	58 (41.1)	45 (32.4)	53 (37.6)
Apprenticeship	128 (30.4)	40 (28.4)	47 (33.8)	41 (29.1)
Secondary school	105 (24.9)	33 (23.4)	31 (22.3)	41 (29.1)
Obligatory education	32 (7.6)	10 (7.1)	16 (11.5)	6 (4.3)
Medication^g^, n (%)				
Yes	127 (30.2)	83 (58.9)	89 (64.0)	77 (55.3)
No	294 (69.8)	58 (41.1)	50 (36.0)	64 (44.7)
Comorbidity^h^, n (%)				
Yes	127 (40.9)	44 (31.2)	46 (33.1)	37 (26.2)
No	294 (59.1)	97 (68.8)	93 (66.9)	104 (73.8)
Diagnosis^i^, n (%)				
Depressive disorder (F32, F33)	204 (48.5)	71 (50.4)	66 (47.5)	67 (48.2)
Anxiety disorder (F40, F41)	66 (15.7)	22 (15.6)	18 (12.9)	26 (18.4)
Reaction to severe stress and adjustment disorder (F43)	83 (19.7)	25 (17.7)	28 (20.1)	30 (20.6)
Other (eg, >10×: F42, F45, F50, F60)	68 (16.1)	23 (16.3)	27 (19.4)	17 (12.8)

a CG: control group.

b *χ*²_4_=0.87; *P*=.93; φ=0.05.

c *χ*²_2_=4.62; *P*=.10; φ=0.1.

d *χ*²_4_=0.44; *P*=.98; φ=0.03.

e *χ*²_8_=10.47; *P*=.23; φ=0.2.

f *χ*²_6_=9.75; *P*=.19; φ=0.1.

g *χ*²_2_=2.01; *P*=.37; φ=0.07.

h *χ*²_2_=2.01; *P*=.37; φ=0.07.

i *χ*²_6_=4.23; *P*=.65; φ=0.10; for diagnosis *ICD-10* (*International Statistical Classification of Diseases, Tenth Revision*) codes are provided.

Descriptive statistics for all measures across all time points (ITT) are displayed in Table 2. Effect sizes reported in Table 2 only were calculated for participants with pre- and follow-up data (completer). All further analyses, unless stated otherwise, were done with the ITT sample.

**Table 2.** Intention-to-treat sample analyses across all instruments and treatment arms^a^.

Measures	Entire sample	REMOTION^b^	Res-Up!^c^	CG^d^^,^^e^
BSI-18^f^				
T1, mean (SD)	1.29 (0.67)	1.32 (0.66)	1.28 (0.67)	1.27 (0.69)
T2, mean (SD)	1.16 (0.70)	1.11 (0.71)	1.21 (0.70)	1.16 (0.70)
T3, mean (SD)	1.07 (0.65)	0.95 (0.62)	1.18 (0.77)	1.07 (0.59)
Within-group ES^g^	0.27	0.34	0.32	0.21
BSI somatization				
T1, mean (SD)	0.87 (0.73)	0.87 (0.77)	0.91 (0.72)	0.82 (0.71)
T2, mean (SD)	0.84 (0.73)	0.77 (0.67)	0.89 (0.76)	0.85 (0.76)
T3, mean (SD)	0.78 (0.64)	0.67 (0.59)	0.92 (0.75)	0.76 (0.59)
Within-group ES	0.16	0.27	0.20	0.07
BSI anxiety				
T1, mean (SD)	1.37 (0.81)	1.40 (0.80)	1.34 (0.77)	1.36 (0.87)
T2, mean (SD)	1.23 (0.83)	1.16 (0.85)	1.28 (0.81)	1.24 (0.83)
T3, mean (SD)	1.13 (0.75)	0.99 (0.70)	1.26 (0.87)	1.13 (0.68)
Within-group ES	0.25	0.28	0.31	0.20
BSI depression				
T1, mean (SD)	1.63 (0.92)	1.68 (0.90)	1.58 (0.91)	1.61 (0.96)
T2, mean (SD)	1.42 (0.92)	1.40 (0.95)	1.47 (0.87)	1.41 (0.93)
T3, mean (SD)	1.30 (0.90)	1.20 (0.88)	1.36 (0.97)	1.31 (0.86)
Within-group ES	0.25	0.28	0.27	0.22
SEK-27^h^				
T1, mean (SD)	50.98 (18.27)	50.09 (17.18)	51.32 (19.85)	51.53 (17.79)
T2, mean (SD)	53.86 (17.75)	56.93 (16.21)	51.85 (17.63)	53.21 (18.59)
T3, mean (SD)	57.61 (20.04)	59.49 (17.84)	60.71 (21.08)	55.06 (20.34)
Within-group ES	−0.27	−0.41	−0.38	−0.15
FrAGe^i^ NE^j^				
T1, mean (SD)	3.07 (0.88)	2.92 (0.86)	3.14 (0.90)	3.16 (0.86)
T2, mean (SD)	3.21 (0.79)	3.18 (0.72)	3.21 (0.91)	3.22 (0.76)
T3, mean (SD)	3.31 (0.92)	3.36 (0.74)	3.40 (1.05)	3.24 (0.92)
Within-group ES	−0.25	−0.48	−0.39	−0.06
FrAGe PE^k^				
T1, mean (SD)	4.45 (1.01)	4.38 (0.99)	4.44 (1.03)	4.52 (1.00)
T2, mean (SD)	4.41 (1.03)	4.54 (0.94)	4.34 (1.14)	4.38 (1.01)
T3, mean (SD)	4.46 (1.05)	4.54 (1.08)	4.34 (1.13)	4.48 (0.99)
Within-group ES	−0.02	−0.11	−0.01	0.02
CD-RISC-10^l^				
T1, mean (SD)	1.68 (0.72)	1.65 (0.69)	1.64 (0.76)	1.75 (0.70)
T2, mean (SD)	1.71 (0.74)	1.68 (0.79)	1.66 (0.76)	1.76 (0.69)
T3, mean (SD)	1.86 (0.75)	1.87 (0.88)	1.84 (0.75)	1.86 (0.70)
Within-group ES	−0.21	−0.25	−0.31	−0.14
WIRF^m^				
T1, mean (SD)	2.78 (0.71)	2.73 (0.69)	2.74 (0.74)	2.86 (0.68)
T2, mean (SD)	2.86 (0.72)	2.89 (0.70)	2.72 (0.80)	2.92 (0.68)
T3, mean (SD)	2.90 (0.79)	2.84 (0.85)	2.89 (0.87)	2.95 (0.71)
Within-group ES	−0.19	−0.19	−0.29	−0.13
PHQ-9^n^				
T1, mean (SD)	12.67 (5.23)	12.91 (5.23)	12.69 (5.28)	12.40 (5.37)
T2, mean (SD)	11.42 (5.50)	10.78 (5.43)	11.84 (5.08)	11.58 (5.77)
T3, mean (SD)	10.74 (5.66)	9.76 (5.43)	11.41 (5.86)	10.82 (5.64)
Within-group ES	0.28	0.43	0.26	0.22
RSES^o^				
T1, mean (SD)	14.28 (7.34)	13.37 (6.87)	14.18 (7.92)	15.28 (7.14)
T2, mean (SD)	14.66 (7.06)	14.64 (7.38)	13.91 (6.89)	15.12 (6.98)
T3, mean (SD)	15.77 (7.20)	16.69 (6.96)	15.36 (7.74)	15.56 (7.03)
Within-group ES	0.08	0.23	0.20	−0.06
SCS-D^p^				
T1, mean (SD)	2.41 (0.59)	2.33 (0.59)	2.43 (0.63)	2.47 (0.55)
T2, mean (SD)	2.44 (0.58)	2.48 (0.57)	2.41 (0.60)	2.43 (0.58)
T3, mean (SD)	2.62 (0.66)	2.64 (0.69)	2.68 (0.69)	2.57 (0.64)
Within-group ES	0.34	0.37	0.47	0.25

a Means and SDs are calculated on the basis of remaining participants at given time points.

b Sample: 42≥n≤141.

c Sample: 47≥n≤139.

d CG: control group.

e Sample: 87≥ n≤141.

f BSI-18: Brief Symptom Inventory-18 [98].

g ES: Cohen’s *d* effect size for pre-post difference of completers calculated using repeated measures ANOVAs; depending on the time point, entire sample: 176≥n≤421.

h SEK-27: Self-Assessment of Emotion Regulation Skills [76].

i FrAGe: Questionnaire Assessing the Acceptance of Unpleasant and Pleasant Emotions [77].

j NE: unpleasant or negative emotion.

k PE: pleasant or positive emotion.

l CD-RISC-10: Connor-Davidson Resilience Scale [79].

m WIRF: Witten Strengths and Resource Form [78].

n PHQ-9: Patient Health Questionnaire-9 [80].

o RSES: Rosenberg-Self-Esteem Scale [81].

p SCS-D: Self-Compassion Scale–German [82].

### Primary Outcome

In the first set of analyses, we focused on between-group differences in overall symptom reduction. Therefore, we examined whether the 3 groups differed in total scores of the primary outcome measure BSI-18, as well as on its 3 subscales—somatization, anxiety, and depression—within the groups and across time (Table 3).

**Table 3.** Results of multilevel model analyses: fixed and random effects of symptom severity (BSI-18)^a^.

	Model 0^b^, *b*^c^ (SE)	Model 1^d^, *b* (SE)	Model 2^e^, *b* (SE)	Model 3^f^, *b* (SE)
Criterion: BSI-18				
Intercept	1.22^g^ (0.05)	1.28^g^ (0.06)	1.26^g^ (0.07)	1.24^g^ (0.07)
Time	—^h^	−0.02^g^ (0.00)	−0.02^g^ (0.00)	−0.01^g^ (0.00)
Group				
CG^i^	—	—	Reference	Reference
REMOTION	—	—	0.05 (0.07)	0.08 (0.08)
Res-Up!	—	—	0.01 (0.08)	0.02 (0.08)
Time×group				
CG	—	—	—	Reference
REMOTION	—	—	—	−0.01 (0.01)
Res-Up!	—	—	—	−0.00 (0.01)
Pseudo-*R*² (fixed/total)	0.00/0.73	0.01/0.75	0.01/0.75	0.02/0.75
ICC^j^				
Patient	0.72	0.73	0.73	0.73
Location	0.02	0.02	0.02	0.02
AIC^k^	1411.55	1377.41^g^	1380.82	1383.72
Cohen’s *d* equivalent^l^				
REMOTION to CG	—	—	—	−0.09
Res-Up! to CG	—	—	—	0.00
Criterion: BSI-18 Somatization				
Intercept	0.84^g^ (0.05)	0.86^g^ (0.05)	0.83^g^ (0.07)	0.82^g^ (0.07)
Time	—	−0.01 (0.00)	−0.01 (0.00)	−0.00 (0.00)
Group				
CG	—	—	Reference	Reference
REMOTION	—	—	0.03 (0.08)	0.06 (0.08)
Res-Up!	—	—	0.07 (0.08)	0.09 (0.08)
Time×group				
CG	—	—	—	Reference
REMOTION	—	—	—	−0.01 (0.01)
Res-Up!	—	—	—	−0.00 (0.01)
Pseudo-*R*² (fixed/total)	0.00/0.70	0.00/0.70	0.00/0.70	0.01/0.71
ICC				
Patient	0.69	0.69	0.69	0.69
Location	0.01	0.01	0.01	0.01
AIC	1551.66	1549.70	1549.85	1552.05
Cohen’s *d* equivalent				
REMOTION to CG	—	—	—	−0.08
Res-Up! to CG	—	—	—	0.00
Criterion: BSI-18 Anxiety				
Intercept	1.29^g^ (0.06)	1.36^g^ (0.07)	1.35^g^ (0.09)	1.34^g^ (0.09)
Time	—	−0.02^g^ (0.00)	−0.02^g^ (0.00)	−0.02^g^ (0.00)
Group				
CG	—	—	Reference	Reference
REMOTION	—	—	0.04 (0.09)	0.06 (0.09)
Res-Up!	—	—	−0.02 (0.09)	−0.01 (0.09)
Time×group				
CG	—	—	—	Reference
REMOTION	—	—	—	−0.01 (0.01)
Res-Up!	—	—	—	−0.00 (0.01)
Pseudo-*R*² (fixed/total)	0.00/0.70	0.01/0.72	0.01/0.72	0.01/0.72
ICC				
Patient	0.69	0.69	0.69	0.69
Location	0.02	0.02	0.02	0.02
AIC	1759.29	1732.00^g^	1735.52	1738.83
Cohen’s *d* equivalent				
REMOTION to CG	—	—	—	−0.07
Res-Up! to CG	—	—	—	0.00
Criterion: BSI-18 Depression				
Intercept	1.53^g^ (0.05)	1.61^g^ (0.06)	1.59^g^ (0.08)	1.58^g^ (0.09)
Time	—	−0.02^g^ (0.00)	−0.02^g^ (0.00)	−0.02^g^ (0.01)
Group				
CG	—	—	Reference	Reference
REMOTION	—	—	0.08 (0.10)	0.10 (0.11)
Res-Up!	—	—	−0.03 (0.10)	−0.02 (0.11)
Time×group				
CG	—	—	—	Reference
REMOTION	—	—	—	−0.01 (0.01)
Res-Up!	—	—	—	−0.00 (0.01)
Pseudo-*R*² (fixed/total)	0.00/0.70	0.01/0.72	0.02/0.72	0.02/0.72
ICC				
Patient	0.70	0.71	0.71	0.71
Location	0.01	0.01	0.01	0.01
AIC	2002.33	1971.75^g^	1974.53	1978.17
Cohen’s *d* equivalent				
REMOTION to CG	—	—	—	−0.06
Res-Up! to CG	—	—	—	0.00

a BSI-18: Brief Symptom Inventory-18 [98].

b Model 0: random intercepts with fixed slopes on the patient level.

c *b*: estimate of predictor of the multilevel regression analysis.

d Model 1: time effect.

e Model 2: between-group effects.

f Model 3: time×group interaction.

g *P*<.001.

h Not applicable.

i CG: control group.

j ICC: intraclass correlation coefficient.

k AIC*:* Akaike Information Criterion. Significance values of AIC indicate results from a likelihood ratio test comparing the current model to the previous model.

l Cohen’s *d* equivalent [93]: effect size.

Results of the MLMs showed that the largest proportion of variation in symptom severity (72.0%) could be attributed to differences between patients, while only 2% could be attributed to differences between locations. In model 1, time was added as a predictor. Symptom severity decreased significantly across the 3 measurements (*b*=−0.02; *P*<.001). The model fit improved significantly (*χ*²_1_=36.1; *P*<.001), resulting in a slightly improved *pseudo-R*² 0.01 for fixed effects and 0.75 for total effects. For the subscales, the model fit improved significantly for anxiety (*χ*²_2_=29.3; *P*<.001) and depression (*χ*²_2_=32.6; *P*<.001), while no significant improvement was found for somatization (*χ*²_1_=7.3; *P*<.01). In model 2, we added the 3 arms (ie, CG, REMOTION, and Res-Up!) to analyze differences between groups. Model fit did not improve (*χ*²_2_=0.6; *P*=.74; subscales: somatization: *χ*²_2_=0.7; *P*=.70; anxiety: *χ*²_2_=0.5; *P*=.79; and depression: *χ*²_2_=1.2; *P*=.54), suggesting that symptom severity at baseline did not differ between the 3 conditions. In the final model (model 3), we examined the time×group interaction to examine whether the change in symptom severity differed between the 3 arms over the course of the study. Model fit did not improve significantly for the GSI (*χ*²_2_=1.3; *P*=.52) or the subscales (all *P* values >.05), suggesting that the change in symptom severity did not differ between the 3 conditions over time. Consequently, our assumption that there is a stronger symptom reduction in the experimental conditions compared to the CG was not supported by these results.

Simple slope analyses for time effects of symptom severity showed significant decrease over time in all 3 groups (all *P*<.006), except for BSI-18 somatization, which did not reach significance in any group (all *P*>.006).

### Secondary Outcomes

#### Emotion Regulation

For the secondary outcome analysis on emotion regulation, we focused on between-group differences in coping with and regulating emotions. Therefore, we examined whether the 3 groups differed in the SEK-27 and the FrAGe scores, with the 2 subscales, acceptance of negative emotions (NE) and acceptance of positive emotions (PE), within the intervention groups and across time.

The results of the MLMs showed that the largest proportion of change in emotion regulation (SEK-27: 69%, FrAGE NE: 63%, and PE: 79%) could be attributed to differences between patients, while no (SEK-27: 0%, FrAGE NE: 0%, and FrAGE PE: 0%) variation in emotion regulation skills could be attributed to differences between locations. In model 1, emotion regulation increased significantly for the SEK-27 (*b*=0.44; *P*<.001) and the FrAGe NE (*b*=0.02; *P*<.001) but not for the FrAGe PE (*b*=−0.01; *P*=.91). Model fit improved significantly for the SEK-27 (*χ*²_1_=28.6; *P*<.001) and the FrAGe NE (*χ*²_1_=21.8; *P*<.001), but not for the FrAGe PE (*χ*²_2_=0.01; *P*=.91). When adding the 3 conditions in model 2 to find differences between groups, model fit did not improve for the SEK-27 (*χ*²_2_=0.1; *P*=.95), the FrAGe NE (*χ*²_2_=3.3; *P*=.19), or the FrAGe PE (*χ*²_2_=0.5; *P*=.77), suggesting that emotion regulation at baseline did not differ between the 3 conditions.

**Figure 4.**
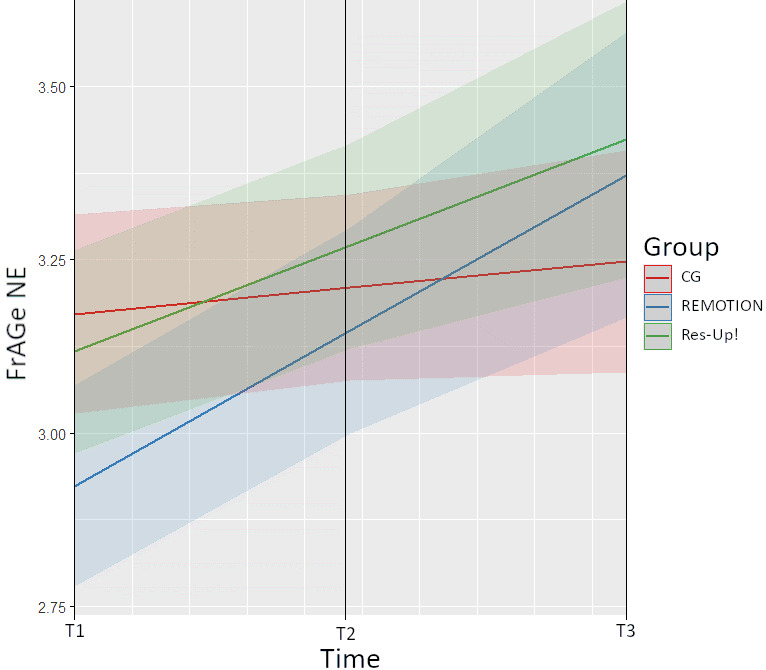
Plot for Questionnaire Assessing the Acceptance of Unpleasant and Pleasant Emotions–Subscale for Unpleasant Emotions (FrAGe NE) across all groups. CG: control group.

In the final model 3, model fit showed no significant improvement for the SEK-27 (*χ*²_2_=5.0; *P*=.08). There were no significant group interaction effects for (1) time×REMOTION (*b*=0.37; *P*=.06) and (2) time×Res-Up! (*b*=0.36; *P*=.07) when compared to time×CG. Regarding FrAGe, model fit did not improve significantly for the subscale FrAGe NE (*χ*²_2_=9.7; *P*=.008), taking into account the adjusted alpha level of .006. For the FrAGe NE subscale, compared with time×CG, we found highly significant differences between time×REMOTION (*b*=0.03; *P*<.001), but not for time×Res-Up! (*b*=0.02; *P*=.06) groups. Model fit did not improve significantly for the FrAGe PE (*χ*²_2_=3.2; *P*=.20). Results for group interactions for the FrAGe PE showed no effect between time×REMOTION (*P*=.09) or time×Res-Up! (*P*=.81) compared to time×CG. Plots are depicted in Figure 4. Consequently, our assumption that there is a stronger increase in the experimental conditions is supported for emotion regulation in REMOTION compared to CG with respect to FrAGe NE only.

Simple slope analyses for time effects showed the following results: SEK-27 improved significantly over time in REMOTION (*P*<.001) and Res-Up! (*P*<.001), but not in the CG (*P*=.05). FrAGe NE showed significant increases in REMOTION (*P*<.001) and Res-Up! (*P*=.002), while changes in the CG were not significant (*P*=.30). FrAGe PE did not show significant change in any group (all *P*>.006).

#### Resilience

For the secondary outcome analysis concerning resilience, we focused on between-group differences in improvements of resilience. Therefore, we examined whether the 3 groups differed in the CD-RISC-10 and the WIRF scores within the intervention groups and across time.

Results of the MLMs showed that the largest proportion of variation in resilience (CD-RISC-10: 75% and WIRF: 68%) could be attributed to differences between patients, while the lesser to none (CD-RISC-10: 0%; and WIRF: 3%) could be attributed to differences between locations. In model 1, resilience increased significantly for both the CD-RISC-10 (*b*=0.01; *P*<.001) and the WIRF (*b*=0.01; *P*<.001). The model fit improved significantly for the CD-RISC-10 (*χ*²_1_=17.7; *P*<.001) and the WIRF (*χ*²_1_=11.9; *P*<.001). In models 2 and 3, no significant changes or interactions could be observed for the CD-RISC-10 (model 2: *P*=.44; and model 3: *P*=.33; interactions compared to time×CG: time×REMOTION, *b*=0.00; *P*=.57; and time×Res-Up!, *b*=0.01; *P*=.13). Model fit did not improve for the WIRF (model 2: *P*=.20; and model 3: *P*=.60; interactions compared to time×CG: time×REMOTION, *b*=0.00; *P*=.52; and time×Res-Up!, *b*=0.01; *P*=.34). Detailed results can be found in Table B1 in Multimedia Appendix 2.

Simple slope analyses for time effects showed the following results: CD-RISC-10 improved significantly only in the Res-Up! group (*P*<.001). Changes in REMOTION (*P*=.04) and the CG (*P*=.06) were not statistically significant at the corrected level. WIRF improved over time in Res-Up! (*P*=.02), but this effect did not reach the corrected α threshold. No significant changes were found in REMOTION (*P*=.05) or the CG (*P*=.12).

### Other Outcomes

#### Depressive Symptoms, Self-Esteem, and Self-Compassion

For further outcome analyses, we focused on between-group differences in reducing depressive symptoms, improving self-esteem, and increasing self-compassion. Therefore, we examined whether the 3 groups differed in the change of scores of the measures PHQ-9, RSES, and SCS within the intervention groups and across time.

The results of the MLMs showed that the largest proportion of variance (PHQ-9: 70%, RSES: 81%, and SCS: 77%) could be attributed to differences between patients, while little to no variation (PHQ-9: 1%, RSES: 0%, and SCS: 0%) could be attributed to differences between locations. There was an overall time effect for the PHQ-9 (model 1: *χ*²_1_=39.3; *P*<.001), but no other effects across all models (model 2: *P*=.86; and model 3: *P*=.16; interactions compared to time×CG: time×REMOTION, *b*=−0.10; *P*=.06; and time×Res-Up!, *b*=−0.01; *P*=.84). No effects were found, taking into account the adjusted α level of .006, for the RSES (model 1: *χ*²_1_=5.3; *P*=.02; model 2: *P*=.21; and model 3: *χ*²_2_=9.5; *P*=.009; interactions compared to time×CG: time×REMOTION, *b*=0.02; *P*=.009; and time×Res-Up!, *b*=0.01; *P*=.01). Overall time effects were found for the SCS (*χ*²_1_=39.7; *P*<.001), but no further effects or interactions (model 2: *P*=.28; model 3: *P*=.10, interactions: all *P* values >.05) could be found. Detailed results can be found in Table B2 in Multimedia Appendix 3.

Simple slope analyses for time effects showed the following results: PHQ-9 significantly decreased over time in all groups: REMOTION (*P*<.001), Res-Up! (*P*=.004), and the CG (*P*<.001), with all values meeting the Bonferroni-corrected significance threshold. RSES results did not meet the corrected significance threshold for REMOTION (*P*=.007) and Res-Up! (*P*=.01), and no significant change was observed in the CG (*P*=.46). SCS significantly increased across all 3 groups (all *P*<.006).

#### Face-to-Face Psychotherapy During the Study

Both REMOTION and CG had a higher rate of parallel regular face-to-face psychotherapy at one (intermittent) or all (2 or 3) of the assessments (Table 4). Answers were categorized as follows: “never,” no concomitant psychotherapy during the whole study; “intermittent,” concomitant psychotherapy at 1 or 2 of 3 time points; and “continuous,” concomitant psychotherapy at all 3 time points.

**Table 4.** Additional psychotherapy during the course of the study.

Therapy	Entire sample (N=421), mean (SD)	REMOTION^a^ (n=141), mean (SD)	Res-Up!^a^ (n=139), mean (SD)	CG^a^^,^^b^ (n=141), mean (SD)
Never	271 (64.3)	89 (63.2)	99 (71.2)	83 (58.8)
Intermittent	91 (21.9)	25 (17.7)	25 (18.0)	41 (29.1)
Continuous	59 (14.0)	27 (19.1)	15 (10.8)	17 (12.1)

a *χ*²_4_ = 11.51 ; *P* = .02 ; φ =0 .17.

b CG: control group.

#### Adherence to the Web-Based Interventions

##### REMOTION

Of all patients randomized to the REMOTION group, 18.4% (*n*=26; Table 5) never started the intervention, despite getting access. On average, participants completed less than half (3 modules) of the program (completed modules, never started=0 modules: mean 2.48, SD 2.12). A module was considered completed if all pages of the module were visited at least once.

**Table 5.** Number of REMOTION and Res-Up! modules completed at follow-up.

Completed modules	REMOTION (n=141), n (%)	Res-Up! (n=139), n (%)	Cumulative (%)	
			REMOTION	Res-Up!
Not started	26 (18.4)	4 (2.9)	18.44	2.88
0	9 (6.4)	27 (19.4)	24.82	22.3
1	27 (19.1)	16 (11.5)	43.97	33.81
2	12 (8.5)	41 (29.5)	52.48	63.31
3	19 (13.5)	12 (8.6)	65.96	71.94
4	16 (11.3)	6 (4.3)	77.3	76.26
5	14 (9.9)	33 (23.7)	87.23	100
6	18 (12.8)	—^a^	100	—

a Not applicable.

##### Res-Up!

Of all patients randomized to the Res-Up! group, 2.9% (n=4; Table 5) never started the intervention, despite getting access. On average, participants completed less than half (2.5 modules) of the program (completed modules, never started=0 modules: mean 2.32, SD 1.82). A module had to be fully filled out and sent in to be considered completed and to gain access to the consecutive module.

### Patients’ Working Alliance

Overall, patients’ ratings of the working alliance stayed the same for REMOTION from T2 to T3 and improved slightly for Res-Up! (Table C in Multimedia Appendix 4). No significant differences between the working alliance ratings in the REMOTION and Res-Up! groups were found in paired *t* tests (all *P* values>.05).

### Reliable Change Index

To assess clinically significant individual-level changes, RCI percentages were calculated for each outcome, with thresholds set at +1.96 and −1.96. Scores were calculated for participants with pre- and follow-up data (for a detailed description, see Table D in Multimedia Appendix 5). For measures where improvement is indicated by symptom reduction (BSI-18 and subscales and PHQ-9), a higher proportion of participants in the CG exhibited reliable improvement compared to the intervention groups. Specifically, 8.5% of CG participants showed reliable improvement on the PHQ-9, compared to 5.7% for REMOTION and 5.8% for Res-Up! Across all BSI subscales and in all groups, deterioration rates remained low, with REMOTION generally showing the lowest rates of reliable worsening.

For outcomes expected to increase with psychological improvement (emotion regulation, resilience, self-esteem, and self-compassion), Res-Up! showed the highest proportion of reliable improvement in resilience (CD-RISC-10; 7.2%) and emotion regulation (SEK-27; 8.6%). The CG exhibited the strongest positive changes in resource use (WIRF; 14.2%), self-compassion (SCS; 12.1%), and self-esteem (RSES; 12.1%). Reliable change was negligible across groups for the FrAGe PE and FrAGe NE subscales, indicating limited effects on the acceptance of positive and negative emotions. Overall, the CG displayed unexpectedly strong rates of reliable improvement on both symptom- and strength-based measures.

## Discussion

### Principal Findings

This study compared 2 transdiagnostic web-based self-help intervention programs, focusing on either emotion regulation as a compensation-oriented intervention, REMOTION, or resilience as a capitalization-oriented intervention, Res-Up!, to a CG. Patients were recruited in routine psychotherapeutic outpatient practice during waiting times. All groups improved significantly over time on symptom severity (BSI-18) as the primary outcome as well as on most secondary outcomes: emotion regulation (SEK-27 and FrAGe) and resilience (CD-RISC-10 and WIRF), as well as on depressive symptoms (PHQ-9) and self-compassion (SCS), but not self-esteem (RSES). Contrary to our hypotheses, we could not find additional improvements of symptom severity, resilience, depressive symptoms, self-esteem, or self-compassion in the intervention groups compared to the CG. Solely concerning one emotion regulation measure, the FrAGe NE, a significantly greater increase of the acceptance of negative emotions was found for REMOTION compared to the CG, with a small effect (*d*=0.21). Further sensitivity analyses of our data are needed to examine additional moderators (eg, face-to-face therapy during the intervention and the number of completed modules) and completer data results.

### Implications

The main result of our study is that the active web-based treatments in our study, REMOTION and Res-Up!, overall did not outperform the CG in most outcomes. Only with respect to REMOTION and the acceptance of NE, our findings suggest that there might be a benefit in participating in web-based interventions for emotion regulation while waiting for psychotherapy. This aligns with findings from the pilot trial, where a sensitivity analysis showed the potential beneficial effects of REMOTION, provided as an add-on to psychotherapy, on emotion regulation [43]. It does not align with previous findings for Res-Up! as a stand-alone intervention on a sample recruited from the general population in German-speaking countries [53], as participants showed significant improvements for resilience (CD-RISC-10 and WIRF), emotion regulation (SEK-27), self-compassion (SCS-D), and self-esteem (RSES).

According to recent literature, focusing on internet-based interventions during waiting time for psychotherapy, that include waiting list CGs, this result pattern of equivalent outcomes in designs with comparisons to a CG seems to prevail [13,99,100]. Our findings align with previous less successful research on popular internet-delivered psychotherapy programs (eg, MoodGYM and Deprexis) during waiting times when compared to care-as-usual [99-101] and especially their difficulties considering implementation in routine care (eg, low uptake, high dropout, and low adherence). Even successful RCTs, where symptoms of depression significantly decreased more in the treatment group (eg, GET.ON Mood Enhancer) compared to a waiting list CG, report significant improvements for waiting list CGs [55]. A meta-analysis on the effects of waiting for face-to-face treatment found changes with small effect sizes in the waiting list CGs of over 29 RCTs, indicating positive influence of being on the waiting list for a treatment compared to not [102]. Participants in our CG were informed that they would get access to one or both programs of their choice after completing all 3 assessments. This might have greatly affected our findings, as the information of being on a waiting list alone can influence the reduction of symptoms or improvement of protective factors [103,104]. Therefore, the significant improvement of the CG across all measures and time points might be due to said factors. Furthermore, our findings might be explained by the heterogeneity within the groups with respect to parallel face-to-face psychotherapy, as it may have influenced the overall process, which could explain why incremental effects of the internet-based treatments were not detected [99]. This is especially relevant because the percentages of people beginning face-to-face therapy highly differed between groups. Access to psychotherapy was not limited at any time during the study, as we did not expect participants to receive therapy so quickly after their first visit to the outpatient centers because of the usually long waiting times in Germany (>3 to 6 mo). As there was no *a priori* control of this factor, the percentage of participants receiving therapy varied greatly across conditions: While only 28.6% of patients in Res-Up! received additional regular psychotherapy at some point or throughout the course of the study participation, 37.1% in REMOTION and 41.4% in the CG did (Table 4).

It has to be taken into account that only half of the eligible patients filled out the baseline questionnaires. This might have been due to different expectations concerning the initial phase of participation. Patients might have not been expecting a questionnaire to be the start of the study participation but a treatment, although they were informed about this by mentioned trained psychotherapists. This might also explain why patients dropped out later, for example, after getting access to a program when they wanted to do the other, or because the transdiagnostic approach did not meet their expectation of a disorder-specific or disorder-focused treatment approach. Recent studies emphasize the importance of individual tailoring of internet-based treatments to increase effectiveness and adherence [105], while others, especially looking at transdiagnostic approaches for anxiety and depression, claim that universal transdiagnostic approaches seem to be superior to tailored ones [19]. Further research is needed to evaluate whether universal or tailored transdiagnostic approaches are more beneficial for treatment outcome as well as adherence and whether that might have been part of the reason for the high dropout rates in this study.

Concerning the question of whether a compensation-focused (REMOTION) or a capitalization-focused (Res-Up!) approach might be more appropriate in an online setting, our findings do not allow an answer, as there were no significant outcome differences between the 2 interventions. In future research, a more comparative approach would be needed in addition to more distinct interventions for compensation and capitalization.

To determine whether web-based interventions during waiting times in routine outpatient care could be effective, more studies are needed, and parallel psychotherapy should be analyzed more closely and in further RCTs.

### Limitations

Several limitations, first in study design and second in intervention design have to be considered. The possibility to generalize our findings to the general population of outpatient psychotherapy patients in Germany is limited because we applied exclusion and inclusion criteria and recruited a selected sample. The trial showed high dropout rates for measurements of 54.3% overall and especially for the treatment groups 64.3% (REMOTION: 67.1%, Res-Up!: 61.4%, and CG: 34.3%), which we tried to accommodate for by pursuing an intention-to-treat-analysis via MLMs and a higher number of participants recruited. Moreover, only 12.8% in REMOTION and 23.7% in Res-Up! completed all modules of the respective intervention, but intervention dropouts were not excluded due to the ITT approach. Additionally, effects of the treatments might have been attenuated further because participants, on average, only completed half of the modules of respective programs (Table 5), which is known to be a substantial influence on the effectiveness [99,100,106]. It is unclear whether patients with less than two-thirds or even half of the treatments completed even received an adequate dosage of the respective treatment’s interventions [100]. While high dropout rates are a frequently reported problem of internet-based interventions [13,54,62], they are likely to be one of the main explanations for our results, for measurement dropouts and especially for the intervention dropouts. Additionally, unguided internet-based self-help interventions without any contact between patient and provider are less effective than therapist-supported internet interventions [64]. As we offered only minimal support and participants had to come forward on their own to receive it, this structure of the internet-based interventions may have impacted the outcomes [59,107]. It may also be possible that we missed occurring longer-term improvements because of the relatively short follow-up period of 12 weeks. Although it might also have led to an even higher dropout rate.

As we did not include sensitivity analyses, in accordance with the ITT approach in the study protocol [56], it limits possible conclusions to our ITT sample and a priori determined outcomes. Additional sensitivity analyses including completer analyses, for measurement points as well as completed modules, or considering parallel access to face-to-face therapy might furthermore have improved the robustness of our analyses and improved the generalizability of our results.

### Future Directions

Results from this study highlight difficulties for implementation and limits of internet-based effectiveness of such interventions during waiting times for psychotherapy. In particular, the study created new knowledge on web-based interventions focusing on resilience and emotion regulation, aligned with capitalization and compensation approaches to psychotherapy. Further analyses may want to examine the interaction of the intervention foci with patient variables: If patients with a particularly high level of adaptive emotion regulation strategies especially benefit from an intervention focusing on the improvement of emotion regulation strategies, it would imply a capitalization change process. If patients with a particularly low level of adaptive emotion regulation strategies benefit particularly from an intervention focusing on the improvement of emotion regulation strategies, this points to a compensation change process. Respective interaction effects between patients’ resilience and a resilience-focused intervention can be hypothesized. Thus, in further analyses, differential effects of patients’ initial levels of resilience and emotion regulation competencies on the outcome of a resilience and emotion regulation intervention should also be explored. Potential limits of internet-based interventions during waiting times are especially present when waiting times are shorter, and there is an overlap of intervention and face-to-face psychotherapy. Therefore, better ways to implement internet-based interventions in the routine setting are direly needed. The results of our study implicate that a focus on more integrated ways of combining such interventions with face-to-face psychotherapy is needed rather than implementing them as stand-alone during patient wait times.

### Conclusions

Therapy Online Plus is a multicenter RCT, comparing a care-as-usual–only CG with two care-as-usual plus 1 of 2 transdiagnostic web-based interventions. One intervention directed at compensating emotion regulation problems—REMOTION—and the other directed at capitalizing on patients’ strengths—Res-Up!—in a clinical sample. Results showed significant improvements across time but no additional benefits of the intervention groups on general symptom severity compared to the CG. Slight benefits of REMOTION on emotion regulation in the form of a higher acceptance of NE compared to CG could be found.

The study extends knowledge on potentials and challenges of implementing transdiagnostic internet-based interventions in routine outpatient psychotherapy settings during waiting times and beyond. Given the time effects found for most outcomes, we can safely assume that both treatments had a positive effect on participants, but no incremental effects when compared to the CG. This has been found in many RCTs for internet-based interventions. Future research should focus on better ways to implement internet-based interventions in routine outpatient care, possible long-term outcomes, and blended care approaches. Furthermore, they will have to evaluate the economic effectiveness and efficiency of transdiagnostic internet-based interventions during waiting times in routine outpatient care. From our findings and a high number of other studies in the field, we have to assume that internet-based interventions, especially transdiagnostic ones, do not seem to be more effective than simply waiting for psychotherapy.

## Supplementary material

10.2196/83917 Multimedia Appendix 1 Assessments listed by time points.

10.2196/83917 Multimedia Appendix 2 Results of multilevel model analyses: fixed and random effects of secondary outcomes.

10.2196/83917 Multimedia Appendix 3 Results of multilevel model analyses: fixed and random effects of other outcomes.

10.2196/83917 Multimedia Appendix 4 Working alliance (WAI-I) of intention-to-treat sample treatment groups.

10.2196/83917 Multimedia Appendix 5 Reliable change index of intention-to-treat sample.

10.2196/83917 Checklist 1 CONSORT checklist.

## Abbreviations

BSI-18: Brief Symptom Inventory-short form
CD-RISC-10: Connor-Davidson Resilience Scale
CG: control group
CONSORT: Consolidated Standards of Reporting Trials
CONSORT-EHEALTH: Consolidated Standards of Reporting Trials of Electronic and Mobile Health Applications and Online Telehealth
FrAGe: Questionnaire Assessing the Acceptance of Unpleasant and Pleasant Emotions
FrAGe NE: Subscale for Unpleasant Emotions
FrAGe PE: Subscale for Pleasant Emotions
*ICD-10*: *International Statistical Classification of Diseases, Tenth Revision*
ITT: intention-to-treat
MLM: multilevel model
PHQ-9: Patient Health Questionnaire-9
PMR: Personal Model of Resilience
RCI: reliable change index
RCT: randomized controlled trial
Res-Up!: Resilienz – unkompliziert und persönlich!
RSES: Rosenberg-Self-Esteem Scale
SCS-D/SCS: Self-compassion Scale – German Version
SEK-27: Self-assessment of Emotion Regulation Skills
WAI-I: Working Alliance Inventory I (adapted for online interventions)
WIRF: Witten Resource Questionnaire
