# Supplementary material for: Evaluation of Two Web-Based Interventions (REMOTION and Res-Up!) for Clients From Psychotherapy Waitlists in Routine Outpatient Psychotherapy (Therapy Online Plus-TOP): Randomized Controlled Trial
Source: J Med Internet Res. 2026 Jul 8;28:e83917. doi: 10.2196/83917 (PMC13345349; doi:10.2196/83917)
Supplement: Multimedia Appendix 1 [file jmir-v28-e83917-s001.docx]

**Table A.** Assessments listed by Time Points

|  |  | Time Point | | |
| --- | --- | --- | --- | --- |
| Variable | Instrument | Baseline | 6 Weeks | 12 Weeks |
| Demographics | | x |  |  |
| Information on current status of psychotherapy | | x | x | x |
| Structured clinical interview / clinical assessment | | x |  |  |
| Primary Outcome | |  |  |  |
| Symptom severity | BSI-18 | x | x | x |
| Secondary Outcomes | |  |  |  |
| Emotion regulation | SEK-27; FrAGe | x | x | x |
| Resilience | WIRF; CD-RISC-10 | x | x | x |
| Depressive symptoms | PHQ-9 | x | x | x |
| Self-esteem | RSES | x | x | x |
| Self-compassion | SCS-D | x | x | x |
| Working Alliance | WAI-I |  | x | x |
| *Note.* BSI-18: Brief Symptom Inventory-18 [71]; SEK-27: Self-assessment of Emotion Regulation Skills [74]; FrAGe: Questionnaire Assessing Acceptance of Unpleasant and Pleasant Emotions [75]; CD-RISC-10: Connor-Davidson Resilience Scale [77]; WIRF: Witten Strengths and Resource Form [76]; PHQ-9: Patient Health Questionnaire-9 [78]; RSES: Rosenberg-Self-Esteem Scale [79]; SCS-D: Self-compassion Scale – German [80]; WAI-I: Working Alliance Inventory Internet interventions [81] | | | | |
