# Supplementary material for: Evaluation of Two Web-Based Interventions (REMOTION and Res-Up!) for Clients From Psychotherapy Waitlists in Routine Outpatient Psychotherapy (Therapy Online Plus-TOP): Randomized Controlled Trial
Source: J Med Internet Res. 2026 Jul 8;28:e83917. doi: 10.2196/83917 (PMC13345349; doi:10.2196/83917)
Supplement: Multimedia Appendix 2 [file jmir-v28-e83917-s002.docx]

**Table B1.** Results of MLM analyses: Fixed and random effects of secondary outcomes

|  | Model 0 | Model 1 | Model 2 | Model 3 |
| --- | --- | --- | --- | --- |
|  | ***b* (SE)** | ***b* (SE)** | ***b* (SE)** | ***b (SE)*** |
| EMOTION REGULATION | | | | |
| Criterion: SEK-27 | | | | |
| Intercept | 1.95^***^ (0.04) | 1.89^***^ (0.02) | 1.89^***^ (0.06) | 1.92^***^ (0.06) |
| Time | / | 0.02^***^ (0.00) | 0.02^***^ (0.00) | 0.01 (0.00) |
| Group | | | | |
| CG | / | / | Reference | Reference |
| REMOTION | / | / | -0.01 (0.08) | -0.06 (0.08) |
| Res-Up! | / | / | 0.02 (0.08) | -0.03 (0.08) |
| TimeXGroup | | | | |
| CG | / | / | / | Reference |
| REMOTION | / | / | / | 0.01 (0.01) |
| Res-Up! | / | / | / | 0.01 (0.01) |
| Pseudo-*R²* (fixed/total) | 0.00 / 0.69 | 0.01 / 0.70 | 0.01 / 0.70 | 0.02 / 0.71 |
| ICC | | | | |
| Patient | 0.69 | 0.70 | 0.70 | 0.70 |
| Location | 0.00 | 0.00 | 0.01 | 0.01 |
| AIC | 1478.74 | 1453.21^***^ | 1457.03 | 1455.92 |
| Cohen’s *d* equivalent |  |  |  |  |
| REMOTION to CG |  |  |  | 0.09 |
| Res-Up! to CG |  |  |  | 0.09 |
| Criterion: FrAGe PE | | | | |
| Intercept | 4.43^***^ (0.05) | 4.44^***^ (0.05) | 4.48^***^ (0.08) | 4.50^***^ (0.08) |
| Time | / | -0.00 (0.00) | -0.00 (0.00) | -0.01 (0.01) |
| Group | | | | |
| CG | / | / | Reference | Reference |
| REMOTION | / | / | -0.08 (0.12) | -0.13 (0.12) |
| Res-Up! | / | / | -0.05 (0.12) | -0.06 (0.12) |
| TimeXGroup | | | | |
| CG | / | / | / | Reference |
| REMOTION | / | / | / | 0.02 (0.01) |
| Res-Up! | / | / | / | 0.00 (0.01) |
| Pseudo-*R²* (fixed/total) | 0.00 / 0.79 | 0.00 / 0.79 | 0.00 / 0.79 | 0.00 / 0.79 |
| ICC | | | | |
| Patient | 0.79 | 0.79 | 0.79 | 0.79 |
| Location | 0.00 | 0.00 | 0.00 | 0.00 |
| AIC | 1969.52 | 1971.51 | 1974.97 | 1975.75 |
| Cohen’s *d* equivalent |  |  |  |  |
| REMOTION to CG |  |  |  | 0.12 |
| Res-Up! to CG |  |  |  | 0.00 |
| Criterion: FrAGe NE | | | | |
| Intercept | 3.14^***^ (0.04) | 3.07^***^ (0.04) | 3.11^***^ (0.07) | 3.17^***^ (0.08) |
| Time | / | 0.02^***^ (0.00) | 0.02^***^ (0.00) | 0.01 (0.01) |
| Group | | | | |
| CG | / | / | Reference | Reference |
| REMOTION | / | / | -0.14 (0.09) | -0.25^**^ (0.10) |
| Res-Up! | / | / | 0.02 (0.09) | -0.05 (0.10) |
| TimeXGroup | | | | |
| CG | / | / | / | Reference |
| REMOTION | / | / | / | 0.03^***^ (0.01) |
| Res-Up! | / | / | / | 0.02 (0.01) |
| Pseudo-*R²* (fixed/total) | 0.00 / 0.62 | 0.01 / 0.64 | 0.02 / 0.64 | 0.02 / 0.65 |
| ICC | | | | |
| Patient | 0.62 | 0.63 | 0.63 | 0.64 |
| Location | 0.00 | 0.00 | 0.00 | 0.00 |
| AIC | 1936.60 | 1916.84^***^ | 1917.54 | 1911.83 |
| Cohen’s *d* equivalent |  |  |  |  |
| REMOTION to CG |  |  |  | 0.21 |
| Res-Up! to CG |  |  |  | 0.14 |
| RESILIENCE | | | | |
| Criterion: CD-RISC-10 | | | | |
| Intercept | 1.72^***^ (0.04) | 1.68^***^ (0.04) | 1.73^***^ (0.07) | 1.75^***^ (0.07) |
| Time | / | 0.01^***^ (0.00) | 0.01^***^ (0.00) | 0.01 (0.00) |
| Group | | | | |
| CG | / | / | Reference | Reference |
| REMOTION | / | / | -0.10 (0.08) | -0.12 (0.09) |
| Res-Up! | / | / | -0.07 (0.08) | -0.10 (0.09) |
| TimeXGroup | | | | |
| CG | / | / | / | Reference |
| REMOTION | / | / | / | 0.00 (0.01) |
| Res-Up! | / | / | / | 0.01 (0.01) |
| Pseudo-*R²* (fixed/total) | 0.00 / 0.75 | 0.01 / 0.76 | 0.01 / 0.76 | 0.01 / 0.77 |
| ICC | | | | |
| Patient | 0.76 | 0.76 | 0.76 | 0.76 |
| Location | 0.00 | 0.01 | 0.01 | 0.01 |
| AIC | 1507.76 | 1492.11^***^ | 1494.46 | 1496.22 |
| Cohen’s *d* equivalent |  |  |  |  |
| REMOTION to CG |  |  |  | 0.00 |
| Res-Up! to CG |  |  |  | 0.08 |
| Criterion: WIRF | | | | |
| Intercept | 2.83^***^ (0.07) | 2.80^***^ (0.07) | 2.88^***^ (0.09) | 2.89^***^ (0.09) |
| Time | / | 0.01 (0.00) | 0.01 (0.00) | 0.01 (0.00) |
| Group |  |  |  |  |
| CG | / | / | Reference | Reference |
| REMOTION | / | / | -0.14 (0.08) | -0.16 (0.08) |
| Res-Up! | / | / | -0.10 (0.08) | -0.13 (0.08) |
| TimeXGroup |  |  |  |  |
| CG | / | / | / | Reference |
| REMOTION | / | / | / | 0.00 (0.01) |
| Res-Up! | / | / | / | 0.01 (0.01) |
| Pseudo-*R²* (fixed/total) | 0.00 / 0.71 | 0.00 / 0.72 | 0.01 / 0.72 | 0.01 / 0.72 |
| ICC |  |  |  |  |
| Patient | 0.68 | 0.69 | 0.68 | 0.68 |
| Location | 0.03 | 0.03 | 0.03 | 0.03 |
| AIC | 1537.73 | 1527.79^***^ | 1528.58 | 1531.57 |
| Cohen’s *d* equivalent |  |  |  |  |
| REMOTION to CG |  |  |  | 0.00 |
| Res-Up! to CG |  |  |  | 0.08 |
| *Note*. Fixed effects are displayed. Model 0: random intercepts with fixed slopes on the patient level; Model 1: time effect; Model 2: between group effects; Model 3: TimeXGroup interaction. Significance values of AIC indicate results from a likelihood ratio test comparing the current model to the previous model. SEK-27: Self-assessment of Emotion Regulation Skills [74]; FrAGe: Questionnaire Assessing Acceptance of Unpleasant and Pleasant Emotions [75], PE: pleasant/positive emotions, NE: unpleasant/negative emotions; CD-RISC-10: Connor-Davidson Resilience Scale [77]; WIRF: Witten Strengths and Resource Form [76];, CG: control group; *b*: estimate of predictor of the multilevel regression analysis, *SE*: Standard Error, *ICC:* Intraclass Correlation Coefficient, *AIC:* Akaike Information Criterion, Cohen’s d equivalent [90]: effect size. * *p*<.006, *** *p*<.001 | | | | |
