# Supplementary material for: Evaluation of Two Web-Based Interventions (REMOTION and Res-Up!) for Clients From Psychotherapy Waitlists in Routine Outpatient Psychotherapy (Therapy Online Plus-TOP): Randomized Controlled Trial
Source: J Med Internet Res. 2026 Jul 8;28:e83917. doi: 10.2196/83917 (PMC13345349; doi:10.2196/83917)
Supplement: Multimedia Appendix 3 [file jmir-v28-e83917-s003.docx]

**Table B2.** Results of MLM analyses: Fixed and random effects of other outcomes

|  | Model 0 | Model 1 | Model 2 | Model 3 |
| --- | --- | --- | --- | --- |
|  | ***b* (SE)** | ***b* (SE)** | ***b* (SE)** | ***b (SE)*** |
| Criterion: PHQ-9 | | | | |
| Intercept | 12.08^***^ (0.40) | 12.57^***^ (0.43) | 12.39^***^ (0.55) | 12.25^***^ (0.56) |
| Time | / | -0.14^***^ (0.02) | -0.14^***^ (0.02) | -0.11^***^ (0.03) |
| Group | | | | |
| CG | / | / | Reference | Reference |
| REMOTION | / | / | 0.28 (0.60) | 0.62 (0.63) |
| Res-Up! | / | / | 0.28 (0.60) | 0.36 (0.63) |
| TimeXGroup | | | | |
| CG | / | / | / | Reference |
| REMOTION | / | / | / | -0.01 (0.05) |
| Res-Up! | / | / | / | -0.01 (0.05) |
| Pseudo-*R²* (fixed/total) | 0.00 / 0.71 | 0.02 / 0.73 | 0.02 / 0.73 | 0.02 / 0.73 |
| ICC | | | | |
| Patient | 0.70 | 0.71 | 0.71 | 0.71 |
| Location | 0.01 | 0.02 | 0.02 | 0.02 |
| AIC | 5058.53 | 5021.26^***^ | 5024.97 | 5025.24 |
| Cohen’s *d* equivalent |  |  |  |  |
| REMOTION to CG |  |  |  | -0.10 |
| Res-Up! to CG |  |  |  | -0.10 |
| Criterion: RSES | | | | |
| Intercept | 1.45^***^ (0.04) | 1.43^***^ (0.04) | 1.50^***^ (0.06) | 1.53^***^ (0.06) |
| Time | / | 0.01 (0.00) | 0.01 (0.00) | -0.00 (0.00) |
| Group | | | | |
| CG | / | / | Reference | Reference |
| REMOTION | / | / | -0.15 (0.08) | -0.20 (0.09) |
| Res-Up! | / | / | -0.06 (0.08) | -0.11 (0.09) |
| TimeXGroup | | | | |
| CG | / | / | / | Reference |
| REMOTION | / | / | / | 0.02 (0.01) |
| Res-Up! | / | / | / | 0.01 (0.01) |
| Pseudo-*R²* (fixed/total) | 0.00 / 0.81 | 0.00 / 0.82 | 0.01 / 0.82 | 0.01 / 0.82 |
| ICC | | | | |
| Patient | 0.81 | 0.81 | 0.81 | 0.81 |
| Location | 0.00 | 0.00 | 0.00 | 0.00 |
| AIC | 1383.60 | 1380.28 | 1381.11 | 1375.64 |
| Cohen’s *d* equivalent |  |  |  |  |
| REMOTION to CG |  |  |  | 0.17 |
| Res-Up! to CG |  |  |  | 0.08 |
| Criterion: SCS-D | | | | |
| Intercept | 2.44^***^ (0.03) | 2.40^***^ (0.03) | 2.42^***^ (0.05) | 2.45^***^ (0.05) |
| Time | / | 0.01^***^ (0.00) | 0.01^***^ (0.00) | 0.01^***^ (0.00) |
| Group | | | | |
| CG | / | / | Reference | Reference |
| REMOTION | / | / | -0.09 (0.07) | -0.12 (0.07) |
| Res-Up! | / | / | 0.01 (0.07) | -0.03 (0.07) |
| TimeXGroup | | | | |
| CG | / | / | / | Reference |
| REMOTION | / | / | / | 0.01 (0.01) |
| Res-Up! | / | / | / | 0.01 (0.01) |
| Pseudo-*R²* (fixed/total) | 0.00 / 0.77 | 0.01 / 0.79 | 0.02 / 0.79 | 0.02 / 0.79 |
| ICC | | | | |
| Patient | 0.77 | 0.79 | 0.79 | 0.79 |
| Location | 0.00 | 0.00 | 0.00 | 0.00 |
| AIC | 1132.55 | 1094.81^***^ | 1096.26 | 1905.72 |
| Cohen’s *d* equivalent |  |  |  |  |
| REMOTION to CG |  |  |  | 0.11 |
| Res-Up! to CG |  |  |  | 0.10 |
| *Note*. Fixed effects are displayed. Model 0: random intercepts with fixed slopes on the patient level; Model 1: time effect; Model 2: between group effects; Model 3: TimeXGroup interaction. Significance values of AIC indicate results from a likelihood ratio test comparing the current model to the previous model. PHQ-9: Patient Health Questionnaire-9 [78]; RSES: Rosenberg-Self-Esteem Scale [79]; SCS-D Self-compassion Scale – German [80]; , CG: control group; *b*: estimate of predictor of the multilevel regression analysis, *SE*: Standard Error, *ICC:* Intraclass Correlation Coefficient, *AIC:* Akaike Information Criterion, Cohen’s d equivalent [90]: effect size. * *p*<.006, *** *p*<.001 | | | | |
