# Supplementary material for: Evaluation of Two Web-Based Interventions (REMOTION and Res-Up!) for Clients From Psychotherapy Waitlists in Routine Outpatient Psychotherapy (Therapy Online Plus-TOP): Randomized Controlled Trial
Source: J Med Internet Res. 2026 Jul 8;28:e83917. doi: 10.2196/83917 (PMC13345349; doi:10.2196/83917)
Supplement: Multimedia Appendix 4 [file jmir-v28-e83917-s004.docx]

**Table C.** ITT intervention sample working alliance (WAI-I)

|  | Total | REMOTION | Res-Up! |
| --- | --- | --- | --- |
| *n* | 83 | 42 | 41 |
| T2, *M* (*SD*) | 2.67 (0.76) | 2.74 (0.83) | 2.60 (0.68) |
| T3, *M* (*SD*) | 2.77 (0.94) | 2.77 (0.96) | 2.77 (0.93) |
| *p* | .23 | .69 | .25 |
| ES | -0.13 | -0.06 | -0.18 |
| *Note.* WAI-I: Working Alliance Inventory Internet version; ES: effect size for the pre–post difference (Cohen’s *d*); Total: sample of REMOTION plus Res-Up! without CG | | | |
