# Supplementary material for: Evaluation of Two Web-Based Interventions (REMOTION and Res-Up!) for Clients From Psychotherapy Waitlists in Routine Outpatient Psychotherapy (Therapy Online Plus-TOP): Randomized Controlled Trial
Source: J Med Internet Res. 2026 Jul 8;28:e83917. doi: 10.2196/83917 (PMC13345349; doi:10.2196/83917)
Supplement: Multimedia Appendix 5 [file jmir-v28-e83917-s005.docx]

**Table D.** ITT sample RCI

|  | RCI % | | | | | | | |
| --- | --- | --- | --- | --- | --- | --- | --- | --- |
|  | Entire Sample | | REMOTION | | RES-UP | | CG | |
|  | < -1.96 | > 1.96 | < -1.96 | > 1.96 | < -1.96 | > 1.96 | < -1.96 | > 1.96 |
| BSI-18 | 7.6 | 1.7 | 7.1 | 0.7 | 7.9 | 2.2 | 8.5 | 2.1 |
| BSI somatization | 6.2 | 2.9 | 5.0 | 0.7 | 5.8 | 2.9 | 7.8 | 5.0 |
| BSI anxiety | 7.6 | 2.1 | 7.1 | 1.4 | 6.5 | 2.9 | 9.2 | 2.1 |
| BSI depression | 7.4 | 2.1 | 5.0 | 2.1 | 7.9 | 1.4 | 9.2 | 2.8 |
| SEK-27 | 4.0 | 8.8 | 3.5 | 9.2 | 2.9 | 8.6 | 5.7 | 8.5 |
| FrAGe PE | 0.2 | 1.0 | 0.0 | 0.0 | 0.0 | 0.0 | 0.0 | 0.0 |
| FrAGe NE | 0.5 | 1.4 | 0.0 | 1.4 | 0.0 | 2.2 | 1.4 | 0.7 |
| CD-RISC-10 | 1.7 | 5.9 | 2.1 | 5.0 | 1.4 | 7.2 | 1.4 | 5.7 |
| WIRF | 6.2 | 10.0 | 2.8 | 6.4 | 3.6 | 9.4 | 12.1 | 14.2 |
| PHQ-9 | 6.7 | 1.9 | 5.7 | 1.4 | 5.8 | 2.9 | 8.5 | 1.4 |
| RSES | 5.0 | 5.2 | 0.7 | 5.0 | 1.4 | 3.6 | 12.1 | 5.7 |
| SCS | 1.7 | 9.5 | 0.7 | 7.1 | 0.7 | 10.1 | 3.5 | 12.1 |
| *Note.* BSI-18: Brief Symptom Inventory [73]; SEK-27: Self-assessment of Emotion Regulation Skills [74]; FrAGe: Questionnaire Assessing Acceptance of Unpleasant and Pleasant Emotions [75], PE: pleasant/positive emotions, NE: unpleasant/negative emotions; CD-RISC-10: Connor-Davidson Resilience Scale [77]; WIRF: Witten Resource Questionnaire [76]; PHQ-9: Patient Health Questionnaire-9 [78]; RSES: Rosenberg-Self-Esteem Scale [79]; SCS-D Self-Compassion Scale – German [80]; RCI: Reliable Change Index, with ITT sample percentage of significant decrease and increase; CG: control group | | | | | | | | |
